# Near-Infrared Spectroscopy to Assess Covert Volitional Brain Activity in Intensive Care

**DOI:** 10.1007/s12028-025-02301-5

**Published:** 2025-06-23

**Authors:** Pardis Zarifkar, Matthew Kolisnyk, Marwan H. Othman, Melika Hassani, Karen Irgens Tanderup Hansen, Morten Hylander Møller, Kirsten Møller, Christine Sølling, Jens Christian Nilsson, Sigurdur Thor Sigurdsson, Michael E. Benros, Jack de Jeu, Karnig Kazazian, John Hauerberg, Kåre Fugleholm, Peter F. Birkeland, Tobias S. Andersen, Jesper Kjaergaard, Daniel Kondziella

**Affiliations:** 1 Department of Neurology, Rigshospitalet, Copenhagen University Hospital, Copenhagen, Denmark; 2 Western Institute of Neuroscience, Western University, London, ON Canada; 3 Department of Intensive Care, Rigshospitalet, Copenhagen University Hospital, Copenhagen, Denmark; 4 Department of Clinical Medicine, University of Copenhagen, Copenhagen, Denmark; 5 Department of Neuroanaesthesiology, Rigshospitalet, Copenhagen University Hospital, Copenhagen, Denmark; 6 Department of Cardiothoracic Anesthesia and Intensive Care, Rigshospitalet, Copenhagen University Hospital, Copenhagen, Denmark; 7 Copenhagen Research Center for Biological and Precision Psychiatry, Mental Health Centre Copenhagen, Copenhagen, Denmark; 8 Department of Neurosurgery, Rigshospitalet, Copenhagen University Hospital, Copenhagen, Denmark; 9 Department of Applied Mathematics and Computer Science, Cognitive Systems, Technical University of Denmark, Lyngby, Denmark; 10 Department of Cardiology, Rigshospitalet, Copenhagen University Hospital, Copenhagen, Denmark

**Keywords:** Brain injury, Coma, Cognitive motor dissociation, Consciousness, Neuromonitoring, Prognostication

## Abstract

**Background:**

Detecting covert consciousness in unresponsive patients is challenging. Although functional magnetic resonance imaging and advanced electroencephalography paradigms can identify volitional brain activity, the limited accessibility of these technologies necessitates alternative approaches. Functional near-infrared spectroscopy may provide a portable solution in the intensive care unit. We assessed the feasibility of functional near-infrared spectroscopy with verbal motor commands to detect volitional brain activity in acute disorders of consciousness (DoC).

**Methods:**

Functional near-infrared spectroscopy recordings and clinical assessments were obtained from 50 patients with DoC with acute brain injury, with data analyzed post hoc and visually at the bedside. Twenty healthy volunteers served as controls.

**Results:**

After quality control, data from 19 controls and 36 patients were analyzed. Cortical activation was detected in 18 (96%) controls and 16 (44%) patients. Among 13 minimally conscious patients, volitional activity was found in 8 (62%), whereas 8 (35%) of 23 clinically unresponsive patients showed activation. Volitional brain activity in the latter was associated with higher odds of command following within a week, although it was not statistically significant (odds ratio 3.1, 95% confidence interval 0.7–15.8; *p* = 0.14). Visual bedside analysis showed high specificity (90%) but moderate agreement (κ = 0.4) with post hoc computational analysis.

**Conclusions:**

Functional near-infrared spectroscopy with motor commands can detect volitional brain activity in acute DoC, although data quality issues remain a limitation.

**Supplementary Information:**

The online version contains supplementary material available at 10.1007/s12028-025-02301-5.

## Introduction

Consciousness levels after brain injury are underestimated in up to 40% of patients who appear clinically unresponsive [1]. This inaccuracy arises from the reliance on behavioral assessments, which depend on observable responses such as command following [2]. Modern neuroimaging has shown that residual cognitive abilities undetectable through bedside examinations, including covert consciousness, can be identified by measuring volitional brain activity [2–5]. Mental imagery tasks such as asking patients to imagine playing tennis during functional magnetic resonance imaging (fMRI) or electroencephalography (EEG) have revealed cortical responses indicating command following in 15–25% of clinically unresponsive patients [2, 3, 6–9]. Although transformative for research purposes, both methods have practical limitations [4]. fMRI offers high spatial resolution but is costly, sensitive to motion artifacts and intracranial devices, and logistically challenging [10, 11], whereas EEG is portable and affordable but subject to environmental noise and poor spatial resolution [12].

Functional near-infrared spectroscopy (fNIRS) is a possible alternative [13, 14]. By detecting changes in cortical hemoglobin oxygenation, fNIRS provides a motion-resilient and cost-effective method for identifying task-related brain activity. These attributes make it interesting for bedside assessment in the intensive care unit (ICU). Preliminary mental imagery paradigms have demonstrated the feasibility of fNIRS for detecting volitional cortical activation in acute and chronic disorders of consciousness (DoC) [13, 15]. However, significant gaps remain, as existing studies are limited by small sample sizes, heterogeneous protocols, and retrospective analyses, and the potential for real-time fNIRS analysis to detect covert consciousness at the bedside is unexplored.

To address this, we investigated whether fNIRS combined with verbal commands could detect volitional brain activation across the DoC spectrum. We asked patients to stick out their tongue, which involves a simple motor action that may be understood even with limited cognitive resources and targets a distinct cortical region within the motor homunculus [16, 17]. We investigated clinically unresponsive and low-responsive patients with DoC and healthy controls, and we explored the feasibility of point-of-care fNIRS analysis at the bedside compared with standard computational post hoc analysis.

## Methods

### Study Design and Population

We prospectively enrolled patients aged ≥ 18 years with acute traumatic or nontraumatic brain injury and impaired consciousness from the ICUs of a tertiary referral center (Rigshospitalet, Copenhagen University Hospital). Exclusion criteria were medically induced coma, ongoing epileptic seizures, preexisting neurological impairments such as aphasia, insufficient Danish or English language proficiency, and physical barriers to cap placement. Unmatched healthy controls were recruited from the local community on a convenience basis.

### Clinical Assessments

Supervised by an experienced critical care neurologist (DK), a neurology resident (PZ) examined patients using the Glasgow Coma Scale [18], the Full Outline of Unresponsiveness [19], and the Simplified Evaluation of Consciousness Disorders [20]. Patients were classified as clinically unresponsive (coma, unresponsive wakefulness syndrome) or clinically low-responsive (minimally conscious state [MCS] negative or positive or better), as outlined in Methods S1 and previous publications [5, 21–24]. Clinical assessments of consciousness levels to document possible recovery of clinical command-following abilities were repeated by the study team for 1 week following fNIRS analysis. Cerebral Performance Category and modified Rankin Scale scores, as well as 3-month outcomes, were ascertained by chart review. All treatment decisions (including withdrawal of life-sustaining therapy) were at the discretion of the attending medical team.

### Classification of Sedation Levels

Sedation levels were recorded and categorized as minimal (absence of intravenous sedation or use of low-dose oral sedatives), moderate (fentanyl < 500 µg/h, remifentanil < 1,000 µg/h, propofol < 100 mg/h, midazolam < 10 mg/h, or sevoflurane < 3%), or high (propofol ≥ 100 mg/h, fentanyl ≥ 500 µg/h, remifentanil ≥ 1,000 µg/h, midazolam ≥ 10 mg/h, sevoflurane ≥ 3%, or any dose of sodium thiopental), as previously described [5, 21–24].

### fNIRS Data Acquisition

**Fig. 1:**
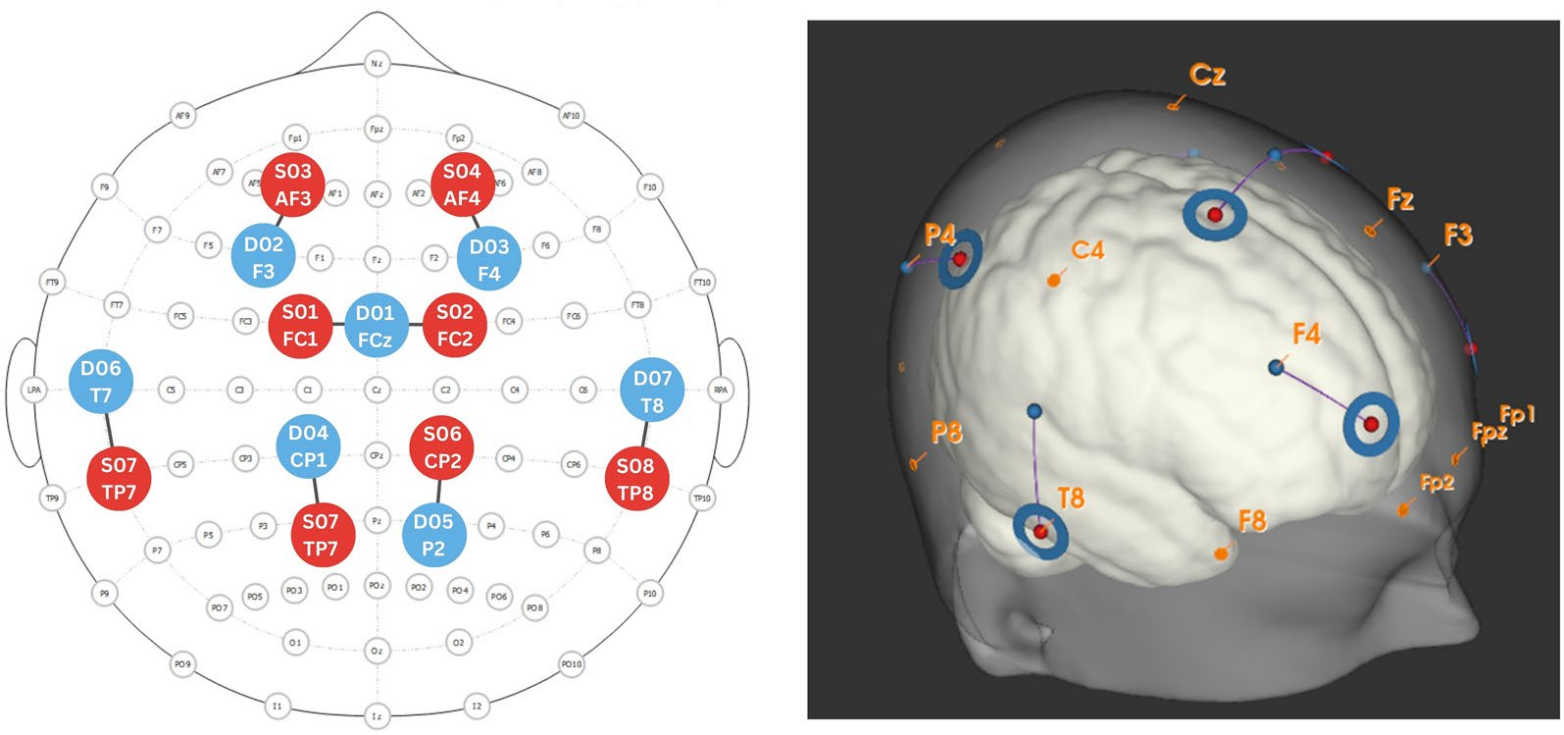
Montage for functional near-infrared spectroscopy data acquisition. The left panel displays a topographic optode layout on a standard 10–10 coordinate system, in which red circles represent sources (S01–S08) and blue circles represent detectors (D01–D07). Optodes were positioned over the frontal cortex, supplementary motor areas, tongue motor homunculi, and parietal cortex, with short-channel detectors placed directly under each source. The right panel shows a 3D reconstruction of the optode placement on a head model, illustrating the spatial arrangement of sources and detectors. Illustrations created in NIRSite and Aurora systems, shown with permission by NIRx Medical system, Berlin, Germany

Continuous wave fNIRS was acquired with Aurora software and a NIRSport 2 system (NIRx Medical Systems, Berlin, Germany). The latter included eight sources and 15 detectors, of which 8 were short-channel detectors, placed according to the international 10–10 coordinate system over the frontal cortex, supplementary motor area, tongue motor homunculi, and parietal cortex (Fig. 1) [16, 17]. Source-detector distances were 3 cm for regular and 0.8 cm for short channels. Data were sampled at 10.17 Hz at wavelengths of 760 nm and 850 nm to capture changes in oxygenated hemoglobin (HbO) and deoxygenated hemoglobin (HbR), respectively.

### Tongue Motor Commands

Tongue motor command tasks consisted of three blocks, each comprising eight repetitions of 15 s tongue movement and 15 s of rest. Healthy controls were instructed to “imagine sticking out your tongue” and patients to “stick out your tongue” (i.e., the patients were asked to actually try to perform the movement). Both were instructed to “stop and rest” in between. The 8 × 3 trial design was considered a reasonable compromise between the number of repetitions required for statistical evaluation on the one hand and the necessity of avoiding participant fatigue on the other. Patients with DoC were assessed during up to three separate sessions over their ICU stay to account for potential fluctuations in arousal, whereas controls underwent a single recording.

### Data Preprocessing

**Fig. 2:**
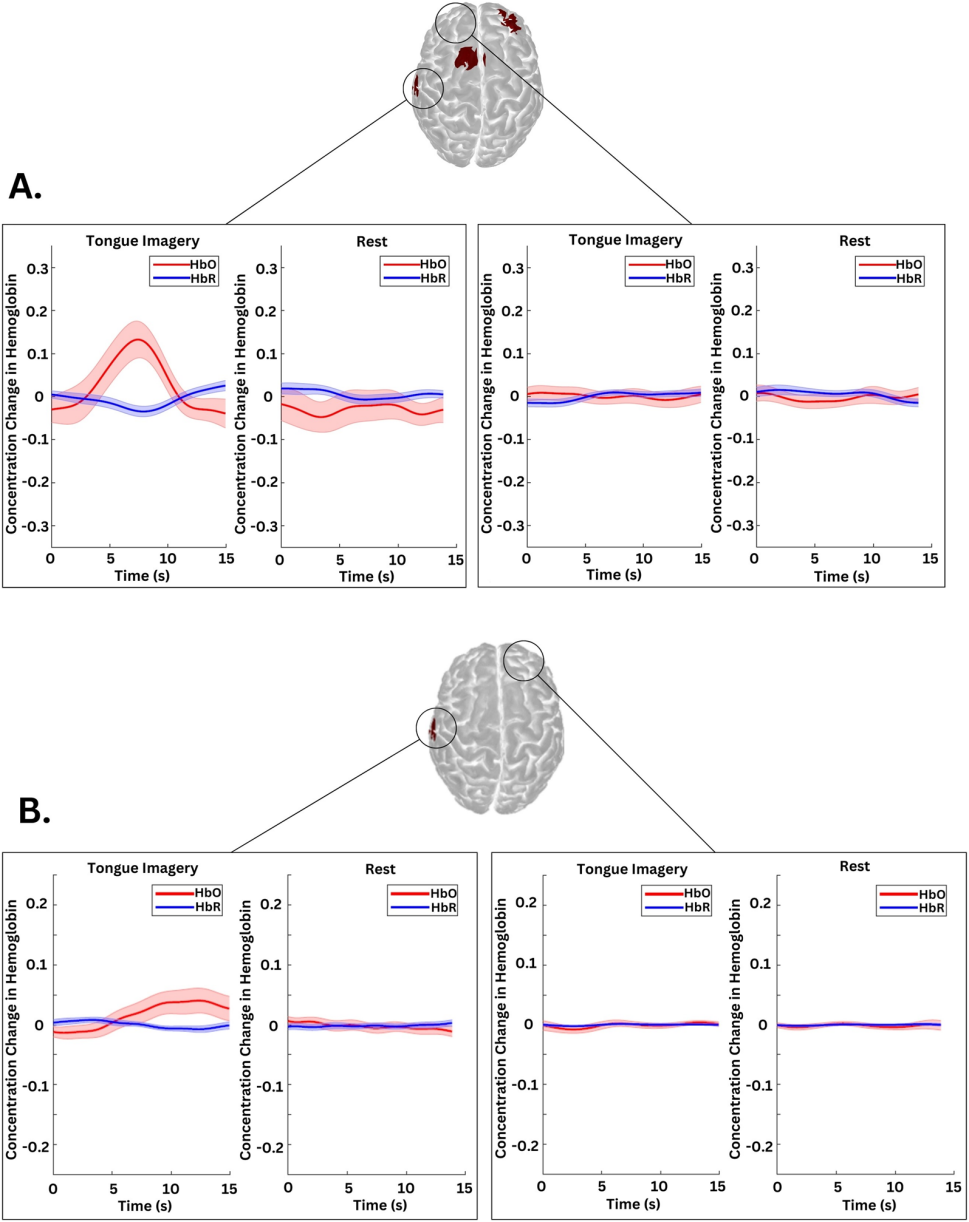
Functional near-infrared spectroscopy time series of two study participants demonstrating volitional brain activity. Three-dimensional cortical maps show task-related cortical activation during a motor task in a healthy control (**a**, identifier [ID] 13 in Fig. 1) and a clinically comatose patient (**b**, ID 7 in Fig. 2). Time series plots display changes in oxyhemoglobin (HbO) (red) and deoxyhemoglobin (HbR) (blue) concentrations across task (tongue motor command) and rest blocks. In the tongue motor homunculus, tongue motor command blocks show a pattern of increased HbO and decreased HbR, consistent with volitional activation (the first block from the left), whereas all other blocks show no significant hemodynamic changes and hence no task-related activations. Shaded areas represent the standard error of the mean

Functional near-infrared spectroscopy data were processed with MATLAB (MathWorks, Natick, MA) and a custom analysis pipeline based on HOMER2 scripts [15, 25]. Raw light intensity signals were converted to optical density values and corrected for motion artifacts using temporal derivative distribution repair methods [26]. Data were then converted to HbO and HbR concentrations (Fig. 2), using the modified Beer-Lambert law [27]. A low-pass filter was applied, using a cutoff of 0.2 Hz determined from analyses of healthy control data to preserve task-related hemodynamic changes while minimizing noise.

To maximize sensitivity in distinguishing between tongue commands and rest conditions, fNIRS preprocessing parameters were optimized by identifying the set that provided the highest between-condition discrimination in the control data set. Grid search was used to evaluate four preprocessing parameters, that is, low-pass filter cutoff = [0, 0.005, 0.01]; high-pass filter cutoff = [0.1, 0.2, 0.3, 0.4, 0.5, 1]; detrending = [TRUE or FALSE], and short-channel regression, using three strategies: (1) adding the spatially nearest short channel as a regressor for each long channel, (2) adding all short channels as regressors for each long channel, and (3) performing principal component analysis on the short channels and using the resulting components as regressors for each long channel. Each combination of preprocessing steps was applied, followed by the same general linear model (GLM) described below to derive test statistics. The objective was to maximize the number of participants with at least one statistically significant channel in the frontal and supplementary motor cortices, excluding parietal regions. The parameter combination yielding the largest number of participants with the expected neural response was considered optimal. Importantly, although this approach uses the control data set both to optimize preprocessing parameters and to evaluate their effectiveness, the patient data set—the study’s primary focus—was not involved in this optimization process. Therefore, the observed sensitivity in the patient data reflects the robustness of this approach.

### Computational fNIRS Analysis

General linear model was employed in patients to assess differences in cortical activation by comparing task-specific hemodynamic response functions during tongue motor commands and rest. Prewhitening was used to address temporal autocorrelation in the signal, and robust regression was applied within the GLM to mitigate the influence of outliers. Channels with insufficient signal quality, indicated by the absence of physiological rhythms such as cardiac activity and respiration, or a signal to noise ratio < 8, were excluded. Moreover, recordings with a larger bad-to-good channel ratio were excluded completely. To remove extracerebral signals arising from cardiorespiratory activity, short channels nearest to the channel of interest were used as regressors to isolate neurovascular activity. Activation patterns during task and rest conditions were quantified using GLM beta coefficients. *p* values from these coefficients were subjected to false discovery rate correction using the Benjamin-Hochberg method [15, 28]. Visualization of spatial maps of activation patterns were generated using Atlas-Viewer, with Monte Carlo simulations to infer the probabilistic path of photons in each source-detector pair, providing a 3D estimate of cortical regions measured by each source-detector pair [29].

### Bedside fNIRS Analysis

Visual assessment of brain activity at the bedside was done for all patients. Prior to task initiation, the 3D cortical activity display was calibrated to baseline by adjusting sensitivity settings to suppress pretask activity, creating a uniform yellow cortical map representing zero activity. During the task, changes in hemodynamic response were visualized as color changes, with red indicating increased activity. Cortical activity was visually inspected during tasks and classified as present when meeting all the following criteria: (1) activation localized to predefined regions of interest, particularly the tongue motor homunculi and supplementary motor area; (2) activation patterns demonstrating temporal consistency with task instructions, allowing for hemodynamic response delays; and (3) activation intensity showing a clear contrast with baseline, marked by robust and sustained red coloration.

### Statistics

Continuous variables are reported as medians with interquartile ranges (IQRs), and categorical variables are reported as frequencies and percentages. To evaluate bedside fNIRS analysis, sensitivity, specificity, and concordance rates were calculated against computational post hoc analysis, and Cohen’s kappa was computed for the level of agreement [30]. Odds ratios (ORs) with 95% confidence intervals (CIs) were used for association between volitional brain activation and recovery of clinical command following. Statistics were performed with R (version 3.6.1).

### Data Availability, Code Sharing, and Ethics Approval

The analysis code is available at https://github.com/TheOwenLab/NCC_Tongue_Imagery, and anonymized data are available on request. Written informed consent was obtained from surrogate decision makers. The regional ethics committee approved the study (H-20026602).

## Results

Between June 2023 and July 2024, we enrolled 50 patients with DoC. Fourteen (28%) patients were excluded due to low-quality fNIRS recordings, resulting in a final cohort of 36 patients (median age 61 years, IQR 16 years; 26 men, 72%) with a total of 46 recordings. At enrollment, 23 (64%) patients were classified as clinically unresponsive (seven in coma, 16 in unresponsive wakefulness syndrome) and 13 (36%) as clinically low-responsive (seven in MCS minus, four in MCS plus, and two emerged from MCS; one of which could produce verbal responses but not follow commands). Primary etiologies were cardiac arrest (33%) and traumatic brain injury (17%). In the control group (*n* = 20), median age was 24 years (± 5 IQR, nine men, 45%); one (5%) participant was excluded due to poor-quality fNIRS. Table 1 and Tables S1–S2 provide clinical details.

**Table 1:** Clinical and demographic characteristics of patients with disorders of consciousness included in functional near-infrared spectroscopy analysis^a^

Characteristic	Total (*N* = 36)	Clinically unresponsive (*n* = 23)^b^	Clinically low-responsive (*n* = 13)^c^
Age, median ± IQR	62 ± 16	60 ± 14	68 ± 19
Sex, male, *n* (%)	26 (72)	17 (74)	9 (69)
Premorbid mRS, *n* (%)			
mRS 0–2	28 (78)	17 (74)	11 (85)
mRS > 2	8 (22)	6 (26)	2 (15)
Charlson Comorbidity Index at baseline, median ± IQR	2 ± 1	2 ± 1	3 ± 2
Cause of ICU admission, *n* (%)			
TBI	6 (17)	4 (17)	2 (15)
Cerebrovascular causes	5 (14)	3 (13)	2 (15)
Other neurological^d^	1 (2)	1 (4)	0 (0)
Cardiac arrest	12 (33)	8 (35)	4 (31)
Other, medical or surgical^d^	12 (33)	7 (30)	5 (38)
GCS score, median (range)	6 (3–15)	5 (3–9)	9 (6–15)
FOUR score, median (range)	8 (2–14)	7 (2–10)	11 (8–14)
ICU admission to enrollment, median ± IQR (d)	8 ± 7	7 ± 9	9 ± 7
Level of sedation during examination, *n* (%)			
None to minimal	25 (69)	17 (74)	8 (62)
Low to moderate	10 (28)	5 (22)	5 (38)
High to very high	1 (3)	2 (4)	0 (0)
ICU survivors, *n* (%)	19 (53)	10 (44)	9 (69)
CPC of ICU survivors at hospital discharge, *n* (%)			
CPC 1–2	11 (58)	5 (50)	2 (46)
CPC > 2	8 (42)	5 (50)	3 (23)
mRS at hospital discharge, *n* (%)			
mRS 0–2	2 (10)	1 (10)	5 (38)
mRS > 2	17 (90)	9 (90)	4 (31)
3-month mortality, *n* (%)	20 (56)	16 (70)	4 (31)

Disorders of consciousness category refers to the level of consciousness at enrollment. CPC, Cerebral Performance Category, eMCS, emerged from MCS, FOUR, Full Outline of Unresponsiveness, GCS, Glasgow Coma Scale, ICU, intensive care unit, IQR, interquartile range, MCS, minimally conscious state, mRS, modified Rankin Scale, TBI, traumatic brain injury, UWS, unresponsive wakefulness syndrome. ^a^See Table S1 for patients excluded from functional near-infrared spectroscopy analysis. ^b^Seven coma patients, 16 patients with UWS. ^c^Seven MCS-negative patients, four MCS-positive patients, two eMCS patients. ^d^See Table S2 for details

### Detection of Volitional Brain Activation in Controls

**Fig. 3:**
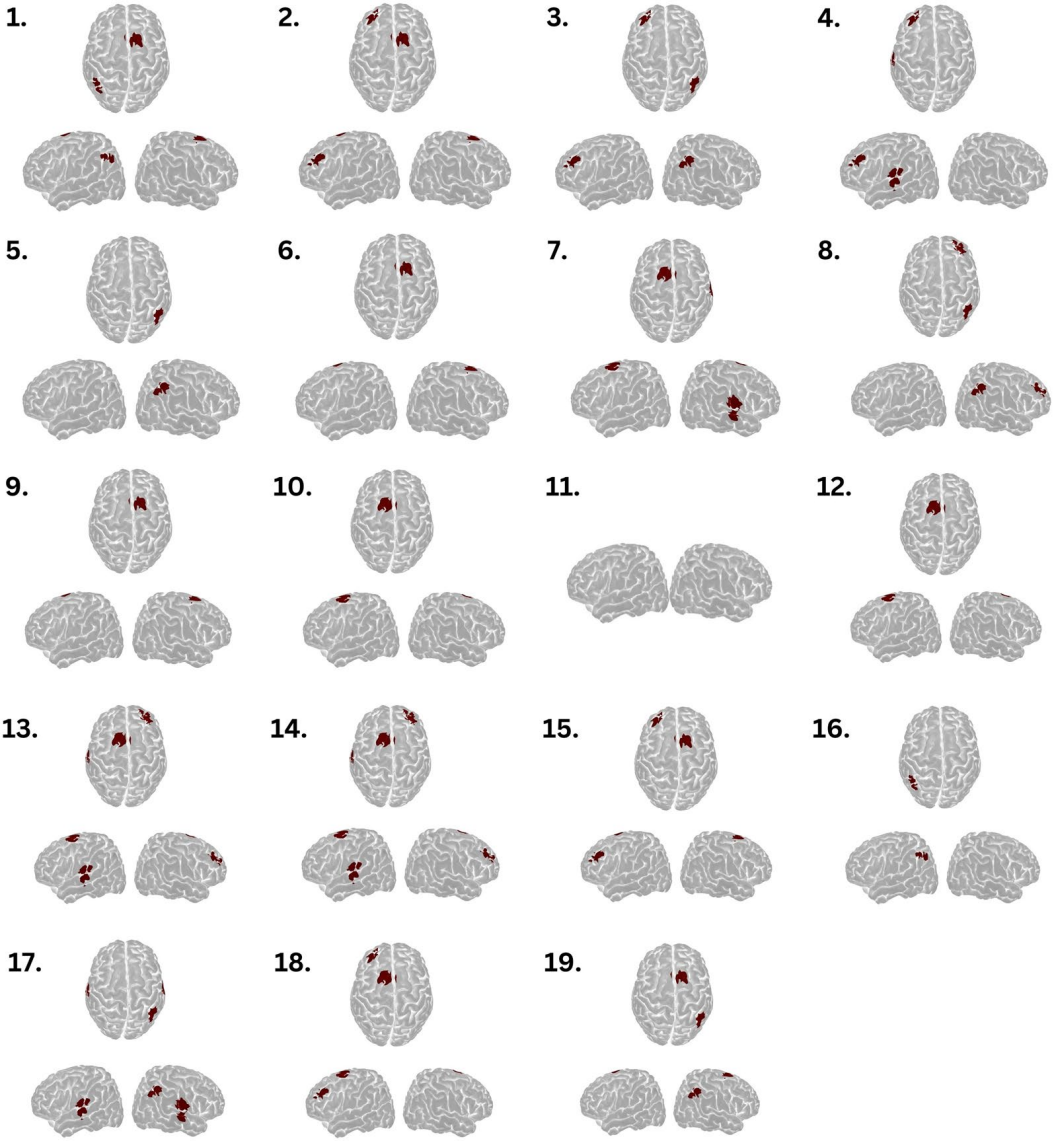
Brain activation patterns in healthy controls during the tongue motor command task. A 3D visualization of cortical regions activated during motor command compared with rest periods from 19 healthy volunteers. Regions highlighted in red indicate channels with significant cortical activation. Activation is predominantly observed in the supplementary motor area, followed by frontal regions and tongue motor cortex

Activity during the tongue motor task was detected in 18 (95%) controls. Cortical activation was most frequent in the supplementary motor area of either hemisphere, followed by frontal regions, tongue motor cortex, and, rarely, posterior parietal cortex (Fig. 3). Table S3 shows HbO and HbR *t*-values.

### Detection of Volitional Brain Activation in DoC

In computational post hoc analysis, fNIRS detected volitional brain activity in 16 (44%) of 36 patients with DoC. Cortical activation was most common in the tongue motor homunculi, followed equally often by the supplementary motor area and frontal regions, and less common in posterior parietal cortex. See Table S4 for HbO and HbR *t*-values.

**Fig. 4:**
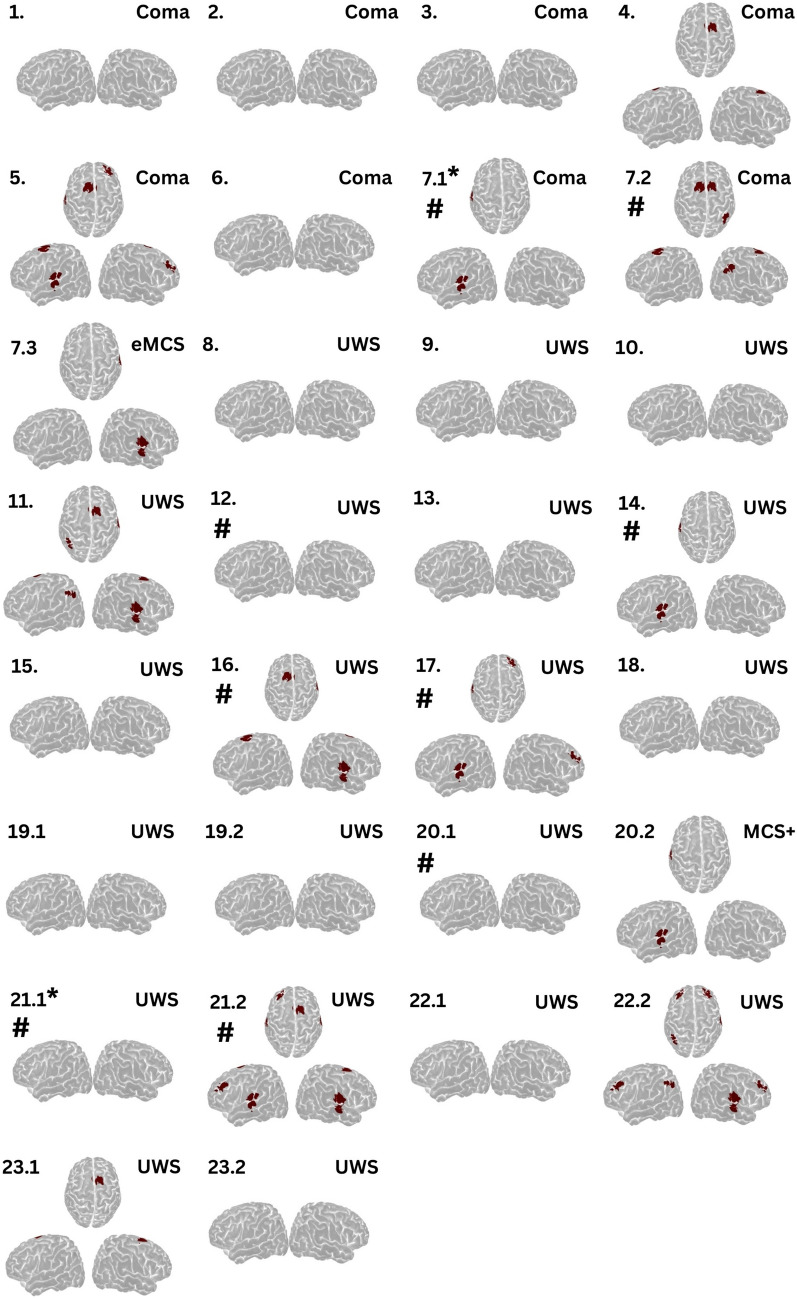
Cortical activation patterns in clinically unresponsive patients during tongue motor commands. Regions highlighted in red indicate significant volitional cortical activation during the task compared to rest, suggesting that these clinically unresponsive patients were in a state of cognitive motor dissociation. Decimal notations indicate the sequence of recordings for patients with multiple assessments. Activation is predominantly observed in the tongue motor cortex, followed by the supplementary motor area and frontal regions. Numbers indicate patient identifiers corresponding to Table S2. *Patients 29 and 36 had progressed to MCS + and eMCS, respectively, during repeated near-infrared spectroscopy evaluation. #Patients who regained command following within 1 week. eMCS, emerging from minimally conscious state, MCS, minimally conscious state, MCS + , minimally conscious state–positive, UWS, unresponsive wakefulness syndrome

**Fig. 5:**
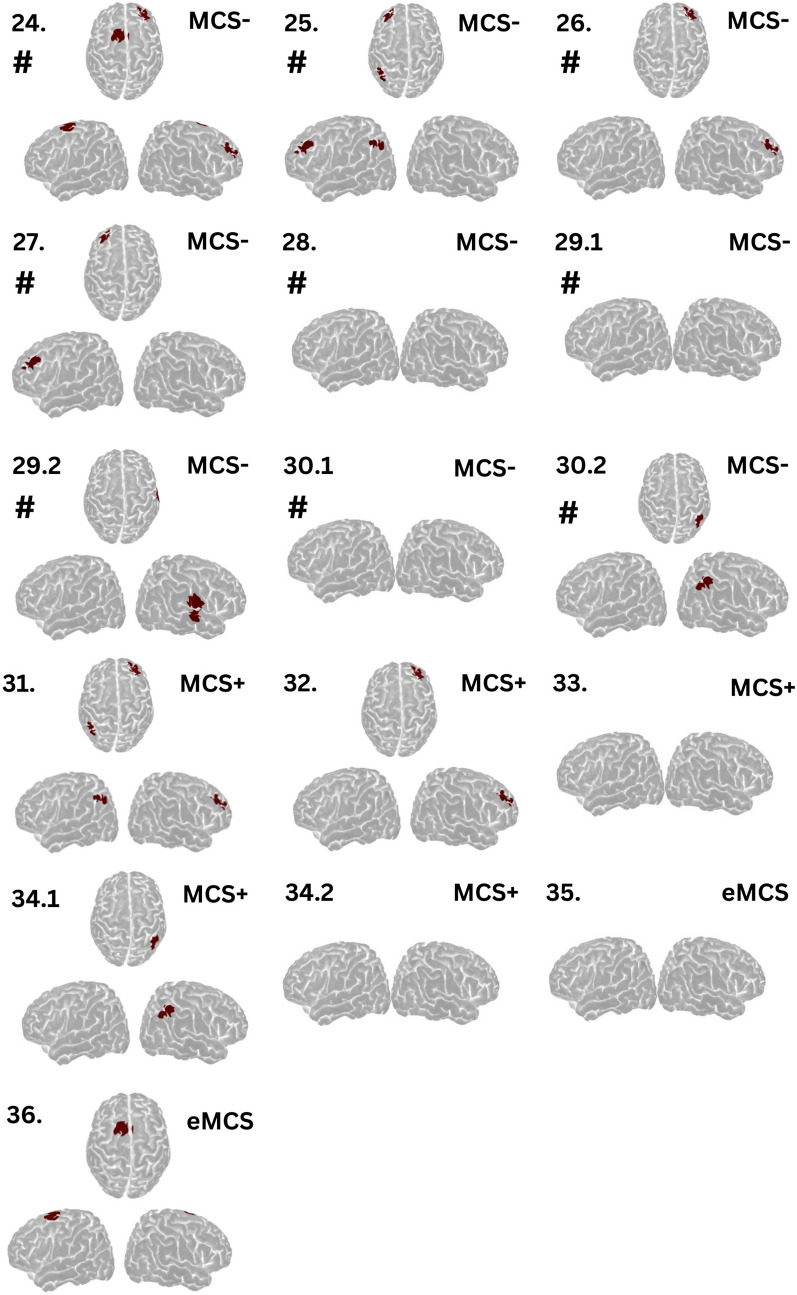
Cortical activation patterns in clinically low-responsive patients during tongue motor commands. Regions highlighted in red indicate significant cortical activation during the task compared with rest. Decimal notations indicate the sequence of recordings for patients with multiple assessments. Numbers indicate patient identifiers corresponding to Table S2. #Patients who regained command following within 1 week. eMCS, emerging from minimally conscious state, MCS, minimally conscious state, MCS − , minimally conscious state–negative, MCS + , minimally conscious state–positive, UWS, unresponsive wakefulness syndrome

Specifically, in 23 clinically unresponsive patients, volitional brain activity was detected in eight (35%, Fig. 4). Presence of cortical activation increased the odds of command following within 1 week, but this was not statistically significant (OR 3.1, 95% CI 0.7–15.8; *p* = 0.14). Furthermore, we identified volitional brain activity during the first examination in 8 (62%) of 13 low-responsive patients (Fig. 5). Of these, four could clinically follow commands, whereas the remaining four regained this ability within 1 week. Among low-responsive patients capable of command following, cortical activation was identified in four (80%), and in seven patients without command following, cortical activation was detected in four (57%). Testing was repeated twice in three MCS-negative patients, which improved the detection rate, with cortical activation identified in six (86%) of seven MCS-negative patients. All these patients progressed to clinical command following within 1 week.

### Comparison of Point-of-Care to Post hoc fNIRS Analysis

Visual fNIRS analyses at the bedside demonstrated moderate agreement with post hoc analyses (concordance rate 72%, Cohen’s kappa 0.4, *p* = 0.01). False negatives were more frequent in clinically unresponsive patients (five of eight patients, 63%). False positives occurred in two clinically low-responsive patients. Specificity was 90%, indicating strong accuracy in identifying absent cortical activation (true negatives), but sensitivity was low (50%).

## Discussion

We show that fNIRS combined with a motor command can detect volitional brain activation across the spectrum of DoC. This activation often precedes observable behavioral recovery, highlighting the potential of fNIRS to identify consciousness recovery.

To our knowledge, only one previous study has investigated fNIRS in acute DoC (*n* = 3 patients), reporting that one ICU patient could intentionally modulate brain activity during a tennis imagery task [15]. Expanding on this work, we applied fNIRS to a larger and more diverse patient cohort, encompassing the full spectrum of DoC. Among clinically low-responsive patients, 62% demonstrated cortical activity, supporting the feasibility of this methodology for detecting volitional brain activity in the ICU. Some patients showed volitional brain activity before regaining the ability to overtly follow commands. This suggests that cortical activation detected with fNIRS may be an early indicator of the potential to recover overt consciousness, consistent with prior studies indicating patients may regain consciousness before they can express it behaviorally [2]. In clinically unresponsive patients, volitional brain activation was identified in 35%. Although the sample size is insufficient to draw conclusions regarding the prevalence of covert consciousness, these findings align with a recent multicenter study that suggests it may be more prevalent than previously assumed [9]. Volitional cortical activation in clinically unresponsive patients was associated with higher odds of command following within 1 week, and although this trend did not reach statistical significance, we believe it indicates a biologically plausible signal.

We also assessed the feasibility of point-of-care fNIRS analysis for detecting volitional brain activity in patients with DoC at the bedside. Although visual analysis demonstrated a high specificity, the sensitivity was low. Visual fNIRS analysis was done by a single investigator, and these results require follow-up studies with more investigators. In sum, future work should focus on refining signal processing algorithms, developing systematic guidelines for signal interpretation, and comparing fNIRS to the diagnostic performance of other technologies such as fMRI [2], EEG [3], and pupillometry [21].

Several limitations must be acknowledged. First, fNIRS measures hemodynamic changes as a proxy for neural activity, which can be influenced by extracerebral signals such cardiac and respiratory rhythms [28]. Although we applied short-channel regression to mitigate these effects, residual extracerebral signal contamination cannot be entirely ruled out. The proportion of fNIRS recordings of insufficient quality was high (> 25%), consistent with well-known methodological challenges of this technology related to, for example, hair color and environmental light [31]. It is therefore not surprising that fNIRS was also unobtainable in one (5%) of the healthy controls. An analogy might be measuring cerebral blood flow through transcranial Doppler; this works in most people, but not in those without a suitable bone window. Second, task adherence is inherently uncertain in DoC. Although the detection of volitional brain activation indicates conscious task engagement, its absence cannot exclude covert awareness. This limitation is particularly relevant in clinically unresponsive patients, when cortical responses may be more subtle, inconsistent, or affected by fluctuating arousal levels. Throughout this article, we have used the term “volitional brain activation” instead of “covert consciousness” to acknowledge that our findings should be regarded as preliminary. Third, the spatial resolution of fNIRS is limited compared with fMRI [32]. Although cortical activation was most frequent in the supplementary motor area in controls and in the tongue motor homunculi in patients, the variability across and within individuals highlights the difficulty of identifying task-specific regions of interest and the need for confirmation by blinded data analysis [15]. Furthermore, our recordings were limited to eight predefined brain regions. High-density fNIRS measurements with whole-head coverage are needed for more comprehensively assessing cortical activation patterns. Moreover, the sample size, although large compared with prior studies [13, 15], remains too small for subgroup analysis. Finally, there are many different fNIRS devices on the market, and each of them require validation.

This study also has strengths. First, we included a relatively large and heterogeneous cohort spanning the full DoC spectrum. Second, we incorporated healthy controls for optimized analysis of fNIRS parameters prior to evaluating the DoC population, reducing the risk of analysis-driven bias, and improving the reliability of cortical activation detection in patients. Third, the present study introduces a tongue motor task, which represents a novel methodology compared with traditional limb-based motor imagery paradigms [15]. This task was selected because it targets a distinct cortical region within the motor homunculus and involves a simple, widely understood motor action. fNIRS in the ICU was convenient, with setup and completion times of approximately 20 min.

## Conclusions

In conclusion, fNIRS combined with tongue motor commands can detect volitional brain activation in the ICU across the entire DoC spectrum. Further research is needed to improve recording quality and to validate fNIRS through “head-to-head” comparison with fMRI and EEG.

## Source of support

This study was funded by Offerfonden, Region Hovedstadens Forskningsfond, Lundbeck Foundation and Rigshospitalets Forskningspuljer. Funding was given to support PhD students and research scholarship students. Open access funding was provided by Royal Danish Library.

## Supplementary Information

Below is the link to the electronic supplementary material.

Supplementary file1 (DOCX 113 KB)
