# Supplementary material for: Near-Infrared Spectroscopy to Assess Covert Volitional Brain Activity in Intensive Care
Source: Neurocrit Care. 2025 Jun 23;44(1):124–35. doi: 10.1007/s12028-025-02301-5 (PMC12819456; doi:10.1007/s12028-025-02301-5)
Supplement: Supplementary file 1 — Supplementary file1 (DOCX 113 KB) [file 12028_2025_2301_MOESM1_ESM.docx]

**SUPPLEMENTARY MATERIAL**

**SUPPLEMENTARY METHODS S1**

**Classification of disorders of consciousness (DoC)**

DoC encompass a spectrum of conditions reflecting varying degrees of impairments in wakefulness and awareness, as outlined below and in ^1^.

| **Disorder of consciousness** | **Definition** |
| --- | --- |
| Coma ^2^ | Coma represents the most severe state, defined by the absence of both wakefulness and awareness.^1^ It is thus a state of profound unawareness, unresponsive to arousal, with an absent normal sleep-wake cycle. Typically lasts a few days to three weeks post-acute brain injury. |
| Vegetative State (VS) / Unresponsive Wakefulness Syndrome (UWS) ^3^ | A state of wakefulness without awareness, where patients may open their eyes but exhibit only reflex behaviors. It is thus characterized by preserved wakefulness without any behavioral evidence of awareness |
| Minimally Conscious State (MCS) ^4,5^ | A state where the patients may exhibit inconsistent yet reproducible non-reflex behaviors in response to environmental stimuli. Patients are classified as MCS if they show signs such as pain localization, visual fixation/tracking, appropriate emotional expressions (MCS minus), or if they can follow commands (MCS plus). |
| Emergence from MCS (eMCS) ^6^ | A transitional state characterized by the recovery of functional communication (e.g., the ability to answer yes/no questions) and/or the use of objects (correctly using at least two different everyday objects). |

**References**

1. Edlow BL, Claassen J, Schiff ND, Greer DM. Recovery from disorders of consciousness: mechanisms, prognosis and emerging therapies. *Nature Reviews Neurology 2020 17:3*. 2020;17(3):135-156. doi:10.1038/s41582-020-00428-x

2. Posner JB, Saper CB, Schiff ND, Claassen J. *Plum and Posner’s Diagnosis of Stupor and Coma*. 5th ed. Oxford University Press, Inc; 2019.

3. Laureys S, Celesia GG, Cohadon F, et al. Unresponsive wakefulness syndrome: A new name for the vegetative state or apallic syndrome. *BMC Med*. 2010;8(1):1-4. doi:10.1186/1741-7015-8-68/PEER-REVIEW

4. Giacino JT, Ashwal S, Childs N, et al. The minimally conscious state: definition and diagnostic criteria. *Neurology*. 2002;58(3):349-353. doi:10.1212/WNL.58.3.349

5. Bruno MA, Vanhaudenhuyse A, Thibaut A, Moonen G, Laureys S. From unresponsive wakefulness to minimally conscious PLUS and functional locked-in syndromes: recent advances in our understanding of disorders of consciousness. *J Neurol*. 2011;258(7):1373-1384. doi:10.1007/S00415-011-6114-X

6. Nakase-Richardson R, Yablon SA, Sherer M, Evans CC, Nick TG. Serial yes/no reliability after traumatic brain injury: implications regarding the operational criteria for emergence from the minimally conscious state. *J Neurol Neurosurg Psychiatry*. 2008;79(2):216-218. doi:10.1136/JNNP.2007.127795

| **Table S1. Clinical and demographic characteristics of DoC patients excluded from fNIRS analysis** | |
| --- | --- |
|  | **Total (n=14; 12 clinically unresponsive and 2 clinically low-responsive)** |
| **Age, median ± IQR** | 64 ± 21 |
| **Sex, male, n(%)** | 6 (43%) |
| **Pre-morbid mRS, n(%)** | |
| mRS, 0-2 | 11 (79%) |
| mRS > 2 | 3 (22%) |
| **Charlson Comorbidity Index at baseline, median ± IQR** | 3 ± 2 |
| **Cause of intensive care unit admission, n (%)** | |
| Cerebrovascular causes | 4 (29%) |
| Other neurological | 2 (14%) |
| Cardiac arrest | 4 (29%) |
| Other, medical or surgical | 4 (29%) |
| GCS score, median (range) | 6 (3-10) |
| FOUR score, median (range) | 8 (1-16) |
| ICU admission to enrolment, days, median ± IQR | 8 ± 8 |
| **Level of sedation during examination, n (%)** | |
| None-minimal | 10 (71%) |
| Low-moderate | 3 (21%) |
| High-very high | 1 (7%) |
| **ICU survivors, n (%)** | 9 (64%) |
| **Cerebral Performance Category of ICU survivors, n (%)** | |
| CPC 1-2 | 5 (56%) |
| CPC > 2 | 4 (44%) |
| **mRS at discharge, n(%)** | |
| mRS 0-2 | 1 (11%) |
| mRS >2 | 8 (89%) |
| **3-month mortality, n (%)^**^** | 8 (62%) |
| DoC category refers to the level of consciousness at enrollment.  * **3 coma, 6 UWS, 3 MCS-, 1 eMCS and 1 lock-in patients**  Abbreviations: CPC, CPC, Cerebral Performance Category; eMCS, emerged from MCS; IQR, interquartile range; MCS, minimally conscious state; mRS, modified Rankin Scale; TBI, traumatic brain injury; UWS, unresponsive wakefulness syndrome.  ** One patient lost to follow-up due to relocation back to home country. | |

**SUPPLEMENTARY RESULTS (TABLES S1-S4)**

| **Table S2. Volitional brain activation and clinical outcomes in DoC patients included in fNIRS analysis** | | | | | | | | | | |
| --- | --- | --- | --- | --- | --- | --- | --- | --- | --- | --- |
| ID | Age | Sex | Cause of brain injury | Days from brain injury to enrollment | Level of sedation | SECONDs score | Tongue motor command: visual analysis | Tongue motor: post-hoc computational analysis | Clinical outcome: 7 days | Clinical outcome: 3 months |
| **Clinically unresponsive patients (coma)** | | | | | | | | | | |
| 1 | 59 | Female | Cerebral microbleeds following lung transplantation | 13 | None to minimal | 0 – No arousal | Not detected | Not detected | Dead | Dead |
| 2 | 68 | Female | Ischemic stroke | 4 | None to minimal | 0 – No arousal | Not detected | Not detected | Dead | Dead |
| 3 | 84 | Male | Cardiac arrest | 3 | None to minimal | 0 – No arousal | Not detected | Not detected | Dead | Dead |
| 4 | 61 | Male | Traumatic brain injury | 2 | None to minimal | 0 – No arousal | Not detected | Detected | Dead | Dead |
| 5 | 52 | Female | Toxic encephalopathy | 9 | None to minimal | 0 – No arousal | Not detected | Detected | Dead | Dead |
| 6 | 63 | Male | Intracerebral hemorrhage | 7 | High to very high | 0 – No arousal | Not detected | Not detected | Unchanged | Alive |
| 7 | 45 | Male | Bickerstaff encephalitis | 6 | None to minimal | 0 – No arousal | Not detected | Detected | Conscious | Alive |
|  |  |  |  | 11 | None to minimal | 0 – No arousal | Detected | Detected |  |  |
|  |  |  |  | 15 | None to minimal | 7 – Intentional  (eMCS) | Detected | Detected |  |  |
| **Clinically unresponsive patients (UWS)** | | | | | | | | | | |
| 8 | 52 | Male | Cardiac arrest | 9 | None to minimal | 1 – Eye opening | Not detected | Not detected | Dead | Dead |
| 9 | 75 | Male | Traumatic brain injury | 8 | Low to moderate | 1 – Eye opening | Not detected | Not detected | Dead | Dead |
| 10 | 55 | Male | Cardiac arrest | 6 | None to minimal | 1 – Eye opening | Not detected | Not detected | Dead | Dead |
| 11 | 69 | Male | Cardiac arrest | 36 | None to minimal | 1 – Eye opening | Not detected | Detected | Dead | Dead |
| 12 | 61 | Male | Hypoxic brain injury (pneumonia) | 32 | None to minimal | 1 – Eye opening | Not detected | Not detected | eMCS | Alive |
| 13 | 23 | Female | Cardiac arrest | 13 | None to minimal | 1 – Eye opening | Not detected | Not detected | Dead | Dead |
| 14 | 62 | Female | Meningitis | 4 | Low to moderate | 1 – Eye opening | Not detected | Detected | eMCS | Alive |
| 15 | 57 | Male | Cardiac arrest | 7 | Low to moderate | 1 – Eye opening | Not detected | Not detected | Unchanged | Dead |
| 16 | 30 | Male | Traumatic brain injury | 14 | None to minimal | 1 – Eye opening | Not detected | Detected | Unchanged | Dead |
| 17 | 62 | Female | Subarachnoid hemorrhage | 27 | None to minimal | 1 – Eye opening | Detected | Detected | MCS+ | Alive |
| 18 | 60 | Male | Cardiac arrest | 2 | Low to moderate | 1 – Eye opening | Not detected | Not detected | Dead | Dead |
| 19 | 51 | Male | Ischemic stroke (basilar artery occlusion) | 1 | Low to moderate | 1 – Eye opening | Not detected | Not detected | MCS- | Dead |
|  |  |  |  | 2 | Low to moderate | 1 – Eye opening | Not detected | Not Detected |  |  |
| 20 | 44 | Male | Traumatic brain injury | 7 | None to minimal | 1 – Eye-opening | Not detected | Detected | eMCS | Alive |
|  |  |  |  | 8 | None to minimal | 7 – Intentional communication | Detected | Detected |  |  |
| 21 | 59 | Male | Ischemic stroke | 4 | None to minimal | 1 – Eye-opening | Not detected | Not detected | MCS+ | Alive |
|  |  |  |  | 5 | None to minimal | 1 – Eye-opening | Detected | Not detected |  |  |
| 22 | 76 | Male | Cardiac arrest | 7 | None to minimal | 1 – Eye-opening | Not detected | Not detected | Dead | Dead |
|  |  |  |  | 8 | None to minimal | 1 – Eye-opening | Detected | Detected |  |  |
| 23 | 69 | Male | Hypoglycemic encephalopathy | 6 | None to minimal | 1 – Eye-opening | Detected | Detected | MCS+ | Dead |
|  |  |  |  | 12 | None to minimal | 1 – Eye-opening | Not detected | Not detected |  |  |
| **Clinically low-responsive patients (MCS-)** | | | | | | | | | | |
| 24 | 76 | Male | Cardiac arrest | 12 | Low to moderate | 5 – Oriented behavior | Detected | Detected | MCS+ | Dead |
| 25 | 64 | Male | Traumatic brain injury | 9 | None to minimal | 4- Visual Pursuit | Detected | Detected | eMCS | Alive |
| 26 | 68 | Female | Anoxic-ischemic brain injury (aorta dissection) | 5 | None to minimal | 4 – Visual Pursuit | Not detected | Detected | eMCS | Dead |
| 27 | 79 | Male | Cardiac arrest | 3 | Low to moderate | 5 – Oriented behavior | Not detected | Detected | eMCS | Alive |
| 28 | 63 | Male | Cardiac arrest | 6 | Low to moderate | 5- Oriented behavior | Detected | Not detected | MCS+ | Alive |
| 29 | 49 | Male | Subarachnoid hemorrhage | 22 | None to minimal | 4 – Visual Pursuit | Not detected | Not detected | MCS+ | Alive |
|  |  |  |  | 23 | None to minimal | 4 – Visual Pursuit | Detected | Detected |  |  |
| 30 | 56 | Male | Traumatic brain injury | 8 | Low to moderate | 4 – Visual Pursuit | Not detected | Not detected | eMCS | Alive |
|  |  |  |  | 9 | Low to moderate | 4 – Visual Pursuit | Detected | Detected |  |  |
| **Clinically low-responsive patients (MCS+)** | | | | | | | | | | |
| 31 | 68 | Male | Septic encephalopathy | 9 | None to minimal | 6 – Command following | Detected | Detected | Unchanged | Dead |
| 32 | 51 | Male | Cardiac arrest | 14 | Low to moderate | 6 – Command following | Detected | Detected | eMCS | Alive |
| 33 | 75 | Female | Acute respiratory distress syndrome | 27 | None to minimal | 7 – Intentional communication | Detected | Not detected | Unchanged | Dead |
| 34 | 53 | Male | Anoxic-ischemic brain injury (aorta dissection) | 12 | None to minimal | 6 – Command following | Detected | Detected | Conscious | Alive |
|  |  |  |  | 13 | None to minimal | 6 – Command following | Not detected | Not detected |  |  |
| **Patients in eMCS** | | | | | | | | | | |
| 35 | 81 | Female | Ischemic stroke | 5 | Low to moderate | 8 – Intentional communication, but no command following | Not detected | Not detected | Conscious | Alive |
| 36 | 73 | Female | Anoxic-ischemic brain injury (aorta dissection) | 5 | None to minimal | 8 – Intentional communication | Detected | Detected | Conscious | Alive |
| DoC category refers to the level of consciousness at enrollment.  Abbreviations: DoC, disorders of consciousness; eMCS, emerged from MCS; MCS, minimally conscious state; SECONDs, Simplified Evaluation of CONsciousness Disorders; UWS, unresponsive wakefulness syndrome. | | | | | | | | | | |

| **Table S3. Cortical activity in the HbO and HbR chromophores during the tongue imagery task in healthy individuals.** | | | | | | | | | | | | | | | | |
| --- | --- | --- | --- | --- | --- | --- | --- | --- | --- | --- | --- | --- | --- | --- | --- | --- |
| **ID** | **Supplementary motor area (L)** | | **Supplementary motor area (R)** | | **Frontal (L)** | | **Frontal (R)** | | **Tongue motor area (L)** | | **Tongue motor area (R)** | | **Posterior parietal (L)** | | **Posterior Parietal (R)** | |
|  | **t-HbO** | **t-HbR** | **t-HbO** | **t-HbR** | **t-HbO** | **t-HbR** | **t-HbO** | **t-HbR** | **t-HbO** | **t-HbR** | **t-HbO** | **t-HbR** | **t-HbO** | **t-HbR** | **t-HbO** | **t-HbR** |
| **1** |  |  | 13.28550559 | -9.659526255 | 4.978335806 | 4.530347948 | -0.074880604 | -1.975783168 | 9.378610101 | 5.693317574 | -3.530315896 | 18.25625516 | 25.03859901 | -14.74568889 | 1.917751514 | -8.280029566 |
| **2** | 5.423041326 | -2.004474434 | 2.742956282 | -9.853847307 | 2.590312225 | -6.649973563 | 0.181888449 | -4.206663472 | -11.67940293 | 1.404016472 | -1.477341559 | 9.008422754 | 2.443502023 | 2.026239757 | 5.42043514 | -0.188642077 |
| **3** | 5.215911224 | 19.40195237 | 1.869171728 | 16.1002869 | 11.34051853 | -10.67982891 | 14.66071373 | 1.0344435 | 16.76518393 | 6.486065855 | -2.066986313 | -5.364076299 | -15.9160712 | -9.380559063 | 8.259125238 | -2.398821479 |
| **4** | 3.21759245 | 4.897130374 | 3.703042304 | 16.22523628 | 2.419398387 | -6.852690277 | 8.540330005 | -0.257976937 | 4.257517488 | -10.66158975 | -8.406908439 | 16.08280173 | -1.835618 | -3.78262967 |  |  |
| **5** | 20.66088948 | 6.03164158 | 21.25519882 | 13.47162089 | 4.478846728 | 7.704905652 | -4.22151875 | -8.317319025 | -1.313023229 | 2.54749294 | -9.206318501 | -3.258782635 | -1.308441172 | 2.393586679 | 6.284252533 | -4.917951495 |
| **6** | 10.60538938 | 6.242728285 | 16.3656927 | -7.102739754 | 1.943840311 | -8.167181834 | -0.736551408 | -6.547803374 | -14.91405759 | 1.053917669 | 10.58571438 | 1.161219849 | -4.643667023 | 4.287839993 | -1.641226202 | 2.916956468 |
| **7** | 12.78536804 | -10.72335599 | -3.239620686 | -4.127656839 | 2.646341805 | 1.193182888 | 0.916396236 | -3.916505139 | 2.276870856 | 23.78932487 | 3.228576856 | -4.067099176 | 18.79804177 | 8.397091264 | 10.63493743 | 9.06602811 |
| **8** | -3.613736292 | 0.447425257 | -1.842446238 | 1.867052899 | 10.78843634 | 5.041972412 | 7.47945863 | -4.273552726 | -4.216334492 | 20.31171645 | -2.259271865 | 6.407389259 | 13.72269105 | 0.529436742 | 10.18121042 | -4.278111602 |
| **9** | 12.02158322 | 8.453838785 | 25.23839612 | -2.693845525 | -0.028916417 | -1.030291738 |  |  | -2.035510249 | 2.777764384 | -2.541584583 | -7.545220659 | -1.764376841 | -6.86818919 | 1.556282337 | -12.43606705 |
| **10** | 14.90790318 | -36.89618157 | 9.223439799 | 11.51033826 | 0.378178606 | 9.408170169 | -8.203555674 | -9.380263259 | 1.702323122 | 8.617232611 | 4.968105496 | 17.29906982 | 11.03895969 | -0.279112709 | -7.742939875 | -0.876276258 |
| **11** |  |  |  |  |  |  |  |  |  |  |  |  |  |  |  |  |
| **12** | 5.204659596 | -6.967200985 | -15.53546171 | 14.64334313 | -5.864022602 | -0.250617336 | -6.971063725 | 15.55387117 | -0.627994833 | -8.884339321 | -9.510215249 | 4.471915558 | 6.191961394 | -0.109928232 | -7.616636941 | 18.08277325 |
| **13** | 41.81133401 | -11.49812451 | -3.727251623 | -13.28613858 | -4.334758015 | 8.386542251 | 2.149365536 | -5.860288259 | 4.019805403 | -2.649532117 | -41.3070978 | -9.024651995 | -9.211741226 | -4.045663933 | -39.80123696 | 15.194442 |
| **14** | 41.81133401 | -11.49812451 | -3.727251623 | -13.28613858 | -4.334758015 | 8.386542251 | 2.149365536 | -5.860288259 | 4.019805403 | -2.649532117 | -41.3070978 | -9.024651995 | -9.211741226 | -4.045663933 | -39.80123696 | 15.194442 |
| **15** |  |  | 2.733469885 | -4.410263427 | 9.730209192 | -3.175956505 | -7.998451094 | -18.32940293 | -7.923629829 | 6.612882269 | 12.31491494 | 0.143682726 | 2.58500507 | 5.244713006 | 2.789249928 | 6.73804536 |
| **16** | 24.49714298 | 17.85739347 | 23.06005384 | 15.46927009 | -18.36386863 | 16.57370255 | -1.138405371 | 11.1961898 | -20.81339412 | 13.57873413 | -18.1623455 | 27.97704096 | 18.39892177 | -41.84256105 | 23.13276017 | 25.93193452 |
| **17** | -6.219978955 | -15.81993291 | 6.825417957 | -0.532750919 | -4.977871457 | -5.952911661 | 0.357495687 | 5.625272246 | 8.939434496 | -3.586518347 | 8.082711724 | -3.29328477 | -4.499217771 | 2.135803246 | 11.32016688 | -6.248553989 |
| **18** | 12.15271706 | -2.748167993 | 13.14041146 | 15.1157929 | 2.202548344 | -5.876079072 | 1.243161157 | -8.34489468 | -1.525440148 | 15.03398833 | -3.038687853 | -2.045359883 | 7.205678194 | 7.117231689 | 4.007756409 | -0.897294164 |
| **19** | 6.016286307 | 6.146977978 | 14.43417989 | -2.740735916 | -5.883151539 | 1.710729395 | -1.409946989 | 11.87165712 | 2.841215939 | -0.493120338 | 4.488281172 | 10.29047674 | -0.086670386 | 4.539797916 | 12.27689886 | -3.922322229 |

| **Table S4. Cortical activity in the HbO and HbR chromophores during the tongue imagery task in patients with disorders of consciousness.** | | | | | | | | | | | | | | | | |
| --- | --- | --- | --- | --- | --- | --- | --- | --- | --- | --- | --- | --- | --- | --- | --- | --- |
|  | **Supplementary motor area (L)** | | **Supplementary motor area (R)** | | **Frontal (L)** | | **Frontal (R)** | | **Tongue motor area (L)** | | **Tongue motor area (R)** | | **Posterior parietal (L)** | | **Posterior Parietal (R)** | |
| **ID** | **t-HbO** | **t-HbR** | **t-HbO** | **t-HbR** | **t-HbO** | **t-HbR** | **t-HbO** | **t-HbR** | **t-HbO** | **t-HbR** | **t-HbO** | **t-HbR** | **t-HbO** | **t-HbR** | **t-HbO** | **t-HbR** |
| **1** | -10.32634581 | -8.836324053 | -4.516791066 | -1.710965311 | -11.67576611 | -5.69573339 | -7.814167093 | -2.159234576 | -8.065013388 | 4.923871315 | -9.320905541 | 4.701581661 | 0.216430542 | 4.55184905 | 0.831791925 | 7.045005105 |
| **2** | 0.002582051 | -22.96591027 | -12.50975942 | -7.209774595 | -5.332042508 | -28.91259642 | -8.802930958 | 4.892287022 | -3.43971488 | 25.28432363 | -8.984537636 | 13.77130786 | -4.741851358 | -14.49455901 | -10.85204114 | 4.081879242 |
| **3** | -2.165584205 | -1.981008747 | 6.462832112 | 12.82334886 | -17.59247103 | -15.05564491 | -0.278550192 | -4.495928165 | -4.566110137 | -6.901428 | -20.77055901 | 1.193527436 | 1.068329875 | 1.336299665 | -1.682009264 | 9.169872608 |
| **4** | -9.921039615 | 11.5336358 | 15.18381906 | -2.715738802 | -4.437237527 | -7.79189056 | -0.872325585 | -22.132967 | 5.934217117 | 2.512117753 | -2.795198564 | -8.156719329 | -2.797271523 | 5.928316396 | -7.708964822 | -12.73048596 |
| **5** | 2.913948595 | -2.478128525 | -7.62751772 | -6.722811104 | -4.202898252 | 2.781802906 | 7.6096858 | -7.681865547 | 5.817790925 | -4.679002875 | 1.095077725 | -2.726904462 | -0.083680621 | -3.589466417 | 3.585972905 | -1.595306323 |
| **6** | -11.68950967 | 7.440306736 | -7.656061162 | -8.767014061 | -1.27092392 | -10.68547892 | -8.721968891 | 9.347995813 | -4.707965237 | 6.807725926 | -7.739834864 | 0.92687191 |  |  | -11.82718963 | -5.308212792 |
| **7.1** | -14.057284 | 10.82122653 | -11.98277752 | 6.750478505 | -3.831349611 | 7.821218647 | -5.279786865 | -4.046615984 | 4.492409862 | -6.446417162 | 1.39836672 | -3.036185473 | -13.04487295 | 20.82482697 | -14.87893771 | 11.03483479 |
| **7.2** | 7.475284156 | -27.56631389 | 3.274256548 | -21.99130152 | -16.63352722 | -11.50689132 | 0.512458803 | 5.546010525 | -24.54892281 | 21.84924281 | -13.06409181 | 11.03983474 | 1.56224312 | -10.14664194 | 18.69028979 | -16.61280707 |
| **7.3** | 0.171308007 | -2.521888258 | 7.026863777 | -0.309240278 | 5.056666481 | 0.35529515 | 6.06719854 | 1.242981998 | -10.93286585 | 19.20036695 | 17.94712781 | -3.644707134 | 9.229501228 | 0.852330838 |  |  |
| **8** | -0.345584072 | 1.620980739 | -5.227827502 | -5.119275464 | 0.157009435 | 13.56607898 | 1.762540619 | -2.352046342 | -17.85332266 | 7.721184581 | 19.57506819 | 16.2835992 | -13.4938843 | 6.979565905 | 10.53234803 | 7.692060161 |
| **9** | -7.529647746 | -13.91066529 | -4.52545003 | -5.008122521 | 9.789022945 | 5.817762546 | -5.368309851 | 9.922112159 | -2.477456618 | -3.37432546 | -1.660288722 | 2.914017045 | 2.004239707 | -0.679169008 | 5.427382341 | 1.548089243 |
| **10** | -2.428332726 | 17.02208068 | -8.90565561 | 8.311923352 | -15.29460512 | 7.782570688 | -6.639804371 | 12.57905936 | -21.91672163 | -4.27465199 | -10.7437982 | -4.436040566 | -12.56263986 | 15.76981668 | -6.652183826 | 17.89900113 |
| **11** | -13.30424131 | 26.34348816 | 21.35619311 | -3.607411505 | -15.61367646 | -10.67740253 | -35.04503542 | -43.33723727 | 4.437043432 | 9.196482726 | 19.28782553 | -6.877789419 | 4.91784374 | -10.76420455 | -21.05780981 | 2.721729075 |
| **12** | -5.534508614 | 7.32693215 |  |  | 7.523106986 | 7.793240638 | 39.85902071 | 3.124482039 | -1.514725853 | 0.796378261 | 6.199434133 | 4.437919623 | -3.546053863 | 0.030138904 | 0.579158732 | -3.612936786 |
| **13** | 1.299970242 | -8.73598499 | -3.233965987 | 4.956732888 | -6.531570542 | -1.954662975 | -20.0649667 | -4.932577053 | 9.858565314 | 9.640320299 | -2.884771375 | -2.218625024 |  |  |  |  |
| **14** | 1.434880236 | 4.487317103 | -1.739857529 | 7.649100473 | 4.874378924 | -3.183062132 | -0.742749026 | 1.899800226 | -6.393342702 | -0.029603421 | -0.199068246 | 6.836716514 | 6.179825166 | 4.489977085 | -3.135200276 | -3.86876792 |
| **15** | 15.28826592 | 21.42976587 | 1.555037544 | 3.988500981 | -18.5233741 | 5.76857829 | -7.902165629 | 22.94043546 | -10.71830794 | 0.231545646 | -24.65727081 | 13.41316003 | -1.160135632 | 0.836736882 | -15.9113946 | 11.44501791 |
| **16** | 10.18723604 | -13.92896542 | 6.053716738 | -1.747593337 | -9.835873364 | 1.626910577 | -12.58910943 | 1.02771933 | -0.380053917 | -6.131615703 | 7.487159423 | -12.75644983 | 1.347944277 | -8.533533916 | -1.090061338 | -1.625883404 |
| **17** | 0.099648932 | 18.7262234 | -1.74760656 | -6.225294418 | 0.498771292 | -2.056817024 | 7.339756872 | -10.9167179 | 5.14092228 | -7.616654745 | 13.19207389 | 1.600090984 | 7.175878492 | 2.402267026 | -2.735482789 | 41.76736091 |
| **18** | -6.051474927 | -1.218225345 | 7.449459104 | 9.598504994 | -0.193284676 | 1.784279894 | -2.774070275 | 3.063108613 | -14.59772848 | 3.462973159 | -11.58669407 | -4.105493491 | 0.280269201 | -7.329742605 | -2.946942782 | 1.15804346 |
| **19.1** | -2.522559445 | -17.53566035 | -18.41414843 | 12.50691769 | -7.113203292 | -16.93804766 | -14.94809328 | -2.459969734 | -7.561891703 | -7.265150701 | -8.977364805 | 8.99210012 | -2.179929541 | -9.957750276 | -7.196349298 | 3.77531518 |
| **19.2** | 13.84550901 | 8.322710079 | 15.71180425 | 13.00139038 | -1.92769652 | 1.895425529 | -6.565777341 | 31.88665747 | -0.511075636 | 0.505420938 | -16.59113505 | 5.883838162 | -8.590675238 | 3.54656969 | -1.287603886 | 0.69256471 |
| **20.1** | -8.006393157 | -7.278312547 |  |  | -1.421770594 | -7.050493573 | -4.522299148 | -2.831182022 |  |  |  |  |  |  |  |  |
| **20.2** |  |  | -2.267651888 | 0.583382973 | -8.660515804 | -5.473106562 | -10.58858022 | -5.277872491 | 7.981836745 | -2.483181756 | 4.115298528 | 5.106975908 | 2.155020624 | 24.59152142 | 13.55084522 | 6.458870445 |
| **21.2** | -17.2676604 | -7.333862885 | -12.74774213 | -0.364664457 | -11.5042208 | 8.376347765 | -7.973216178 | 7.52795759 | -3.379841359 | -7.014643303 | -1.252104193 | -10.72951298 | -7.352572182 | 2.649123269 | 9.227058754 | 5.503964778 |
| **21.2** | 0.94020002 | -4.148125823 | 3.929760666 | -7.285255382 | 8.912273723 | -9.065384424 | 0.45015307 | -3.503833684 | 3.046637072 | -2.436450597 | 13.72722756 | -6.649673869 | -9.325556744 | 3.42154596 | 8.075789046 | 0.795163157 |
| **22.1** | -6.573097888 | 15.95816165 | 4.554824712 | 5.555007997 | 1.483077982 | -2.427145371 | -2.838460813 | 6.398277538 | -5.900363378 | -6.470490986 | -0.721608901 | -11.72499067 | -2.372521938 | 1.77265792 | -21.78833668 | -14.87566433 |
| **22.2** | 22.49198522 | 2.527111483 | 1.884501228 | -11.16468504 | 16.60003801 | -9.817610589 | 40.82726949 | -9.640935733 | -1.129411009 | -9.126753256 | 3.182088583 | -7.931108459 | 14.66529687 | -3.889704681 | 4.066139371 | 2.937944438 |
| **23.1** | -5.316037395 | -15.08807956 | 5.2894526 | -28.02109533 | 10.03131743 | 24.87359567 | -1.357476749 | -11.01479229 | -8.826531064 | 6.089845082 | -6.900816329 | -4.134627686 | -9.851902166 | 2.266263232 | 1.284109064 | 2.888164054 |
| **23.2** |  |  | -11.8304722 | -5.970548864 |  |  | -5.731129318 | -17.89709424 |  |  | 8.101367403 | 3.820767353 | -1.603887017 | -7.058822799 | -13.0054659 | -5.679420279 |
| **25** | 15.12314432 | 21.51338053 | 21.0594072 | 22.16914353 | 15.26524446 | -3.113951702 | 9.137166171 | 30.63514506 | -13.5132959 | 10.24422962 | 17.75811718 | 19.79155637 | 10.40951317 | -24.91964301 | 2.404584877 | 27.91504222 |
| **25** | 2.920957182 | -7.69777398 | -0.932913418 | -7.829600546 | -2.137458613 | 0.219607358 | 4.858253426 | -2.828658619 | -7.164600771 | -12.84057419 | -10.06926005 | -3.427056877 | -7.82783506 | -14.87646062 | -5.235989126 | -3.335342127 |
| **26** |  |  | -0.782509585 | 1.449050286 | -3.812428854 | -4.250725263 | 3.300856282 | -3.558887626 | 2.256345962 | 3.205049391 | -6.50463073 | -12.92372078 |  |  | -4.098833152 | 2.081000294 |
| **27** | 4.942641584 | 8.628591619 | 4.330166524 | 1.057107483 | 5.531153268 | -7.904847166 | -2.881672276 | 6.691624836 |  |  | -1.231257491 | 1.504468782 |  |  |  |  |
| **28** | -5.089919239 | -6.433255872 | -3.297676015 | -0.166367288 | -18.57510547 | -7.608357648 | -9.87181896 | -13.49401495 | -14.76333418 | -15.82511111 | 5.717849982 | 38.2156591 |  |  | -2.00941484 | 11.91045077 |
| **29.1** | 5.260830985 | 4.810202497 | -2.575293478 | -9.986502445 | -10.77237207 | -20.45193447 | 0.442947011 | -7.610897466 | 0.972792128 | 9.919587764 | 7.604415409 | -4.578113808 |  |  | 26.24953399 | 11.89072848 |
| **29.2** |  |  | -18.80163029 | -10.53084208 |  |  | -31.79139202 | 16.68180842 |  |  | -16.68492392 | 4.335102536 |  |  | -20.87498098 | -10.26910851 |
| **30.1** | 11.41163921 | 1.507138497 | 3.67662397 | 13.23280656 | -5.513631524 | 4.562318229 | 3.392775211 | 5.024681753 | 2.076626066 | 5.153562305 | 7.601059684 | -1.54350047 | -3.709716703 | -2.784339445 | -10.51562057 | 6.904831359 |
| **30.2** | -5.815834641 | -1.281338466 | 0.92424002 | 4.970581219 | -1.247737152 | -3.907462805 | 2.488252203 | -0.353038895 | 2.947712199 | -0.539258775 | 2.403299589 | -0.879927407 | 1.542891584 | 6.678659557 | 5.354571494 | -18.03679601 |
| **31** | -8.283329619 | 16.68250696 | -3.981448625 | -2.266632595 | -5.816181395 | -2.881215398 | 6.034003819 | -6.363749647 | -18.96956021 | 5.462496643 | -7.068170842 | 1.205608599 | 2.410804827 | -2.128336548 | -11.75431029 | 4.287658308 |
| **32** | 7.009040057 | 6.546042416 | 3.060615 | 4.791931378 | -11.44463536 | -1.750834567 | 7.55407529 | -4.04175845 | -1.428240443 | 0.249467692 | -2.027406969 | -1.257474634 | -6.805797331 | -4.164851525 | -8.294866587 | 2.820469779 |
| **33** | 0.428495584 | 3.287500325 |  |  | -12.30619941 | 2.614110477 |  |  | 1.321482446 | -2.902761073 |  |  | 0.810766512 | -4.54703413 | -3.404667313 | -1.132898854 |
| **34.1** | -14.86412488 | -11.45477651 | 13.88175311 | 15.59219303 | -15.52109417 | 10.00622709 | 14.5788221 | 1.682521641 | -3.067927692 | 8.316264874 | -9.204039119 | -4.12003742 | -12.39388048 | -9.549609108 | 11.90645466 | -3.456154612 |
| **34.2** | 0.3953785 | 9.072468704 | 3.31091253 | -2.029914121 | -8.483353047 | 0.232965979 | -2.253335924 | -0.754131132 | 13.81196374 | -2.376123129 | -1.0038017 | 4.812474055 | 9.037646324 | 8.008009662 |  |  |
| **35** |  |  |  |  |  |  | 19.87609463 | -0.470332851 | -5.903051935 | 1.205209896 | -5.876279661 | -3.963947923 |  |  |  |  |
| **36** | 8.531219014 | -7.274904576 | -9.532762553 | -4.404033308 | 6.528133396 | 0.819675017 |  |  |  |  | -2.418517981 | 0.206380463 | -17.69708422 | -19.36577842 |  |  |
